# Neutrophil‐to‐lymphocyte ratio is associated with stroke progression and functional outcome in patients with ischemic stroke

**DOI:** 10.1002/brb3.3261

**Published:** 2023-09-24

**Authors:** Chongxi Xu, Linrui Cai, Tong Yi, Xingyang Yi, Yu Hu

**Affiliations:** ^1^ Department of Neurosurgery, West China Hospital Sichuan University Chengdu Sichuan China; ^2^ National Drug Clinical‐Trial institution of West China Second Hospital Sichuan University Chengdu China; ^3^ NMPA Key Laboratory for Technical Research on Drug Products In Vitro and In Vivo Correlation Chengdu China; ^4^ Key Laboratory of Birth Defects and Related Diseases of Women and Children Sichuan University, Ministry of Education Chengdu China; ^5^ Department of Neurology, West China Hospital Sichuan University Chengdu Sichuan China; ^6^ Department of Neurology People's Hospital of Deyang City Deyang Sichuan China

**Keywords:** functional outcome, ischemic stroke, neutrophil‐to‐lymphocyte ratio, stroke progression

## Abstract

**Objective:**

The objective of the present research was to examine the correlation between the neutrophil‐to‐lymphocyte ratio (NLR) and stroke progression (SP) as well as the functional outcome following an ischemic stroke (IS).

**Methods:**

The current study was conducted as prospective observational research. A cohort of 341 participants diagnosed with IS was included in the study from March 2019 to August 2021. This study's primary measure of interest was the occurrence of SP within the initial week following hospital admission. The secondary outcome was functional status 3 months after IS as measured by a modified Rankin scale score. The association between NLR with SP, and poor functional outcomes was examined using multivariate logistic regression. The predictive value of NLR for SP and poor functional outcomes was evaluated using the receiver operating characteristic (ROC) curve.

**Results:**

Among the 341 enrolled patients, 56 (16.4%) had SP, and 285 (83.6%) had no SP. The results of the multivariate logistic regression analysis demonstrated that the existence of diabetes mellitus and the NLR were independently associated with SP and poor functional outcomes. The area under the ROC curve of NLR in predicting poor functional outcome was 0.6117 (95% confidence interval, .5341–.6893, *p* = .0032), and the optimal cut‐off point was 4.2139. The sensitivity and specificity of NLR in predicting poor functional outcomes were 52.7% and 72.0%, respectively.

**Conclusion:**

Patients with acute IS exhibited a very high incidence of SP. NLR may be a valuable prognostic indicator in clinical practice because it was independently associated with SP and a poor functional outcome.

## Graphical abstract

1 Diabetes mellitus and the upregulated neutrophil‐to‐lymphocyte ratio were associated with presence of stroke progression in acute ischemic stroke patients during hospitalization. 2 Upregulated neutrophil‐to‐lymphocyte ratio is significantly associated with 3‐month poor outcome within ischemic stroke cases. 3 Stemming from this investigation.s results, the occurrence of stroke progression seems unrelated to the territory or types of ischemic stroke.

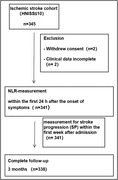

## 1 BACKGROUND

Ischemic stroke (IS) is a prevalent mortality leader within China, together with functional impairment (Meschia et al., 2014; Yang et al., 2013). Most individuals with acute IS often encounter a condition known as early neurological deterioration (END) shortly after being admitted to the hospital. The occurrence of complications, such as progressive stroke (SP), bleeding, edema, and high intracranial pressure, during the end stage of acute stroke is an important aspect that is closely associated with unsatisfactory functional outcomes (Kwan & Hand, 2006). Previous literature found that SP is one of the most frequent causes of END (Weimar et al., 2005). The etiology and pathogenesis of SP remain unclarified as its definition is still non‐standardized (Birschel et al., 2004). According to previous research and our findings, SP was defined in this study as a two‐point increase in the overall National Institute of Health Stroke Scale (NIHSS) score in the first week after admission, with the exclusion of hemorrhagic transformation (HT), novel IS in other areas, and edema (Kwon et al., 2014).

Inflammation and immune systems have been identified as critical components of IS pathogenesis (Macrez et al., 2011). Peripheral cells, such as neutrophils, have been associated with an increased risk of IS (Zhu et al., 2018), and they play critical roles in SP and in‐hospital mortality (Kazmierski et al., 2004). Monocytes can be prognostic predictors in IS cases (Urra et al., 2009). T lymphocyte subtypes exacerbate brain damage after IS (Meng et al., 2019). Many studies have demonstrated a correlation between various inflammatory composite indicators and ISs. These indicators include the neutrophil‐to‐lymphocyte ratio (NLR) (Tokgoz et al., 2013), platelet‐to‐lymphocyte ratio (PLR), lymphocyte‐to‐monocyte ratio (LMR) (Lux et al., 2020), and monocyte‐to‐high‐density lipoprotein ratio (MHR) (Wang et al., 2019). However, the literature lacks sufficient reporting on the association between these inflammatory composite markers and SP. The potential association between NLR and the development of IS complications, such as systemic inflammation and SP, has yet to be conclusively established. Therefore, this study aimed to examine the potential connections between inflammatory composite indicators and SP and outcomes at a 3‐month follow‐up in a cohort study. This study also analyzed the possible correlation between NLR levels and a newly proposed stroke subtype classification (Han et al., 2007).

## 2 METHODS

### 2.1 Study cohorts

A total of 341 individuals diagnosed with first‐ever IS were included in this study, with enrollment consecutively between March 2019 and August 2021. The participants were recruited from two medical institutions, the People's Hospital of Deyang City and the Second People's Hospital of Deyang City. Patients with IS were confirmed via brain magnetic resonance imaging (MRI) scan and were admitted to the participating hospitals within the first 24 h after the onset of symptoms.

Inclusion criteria: (1) age ≥50 years; (2) NIHSS score ≤10 on admission.

**FIGURE 1:**
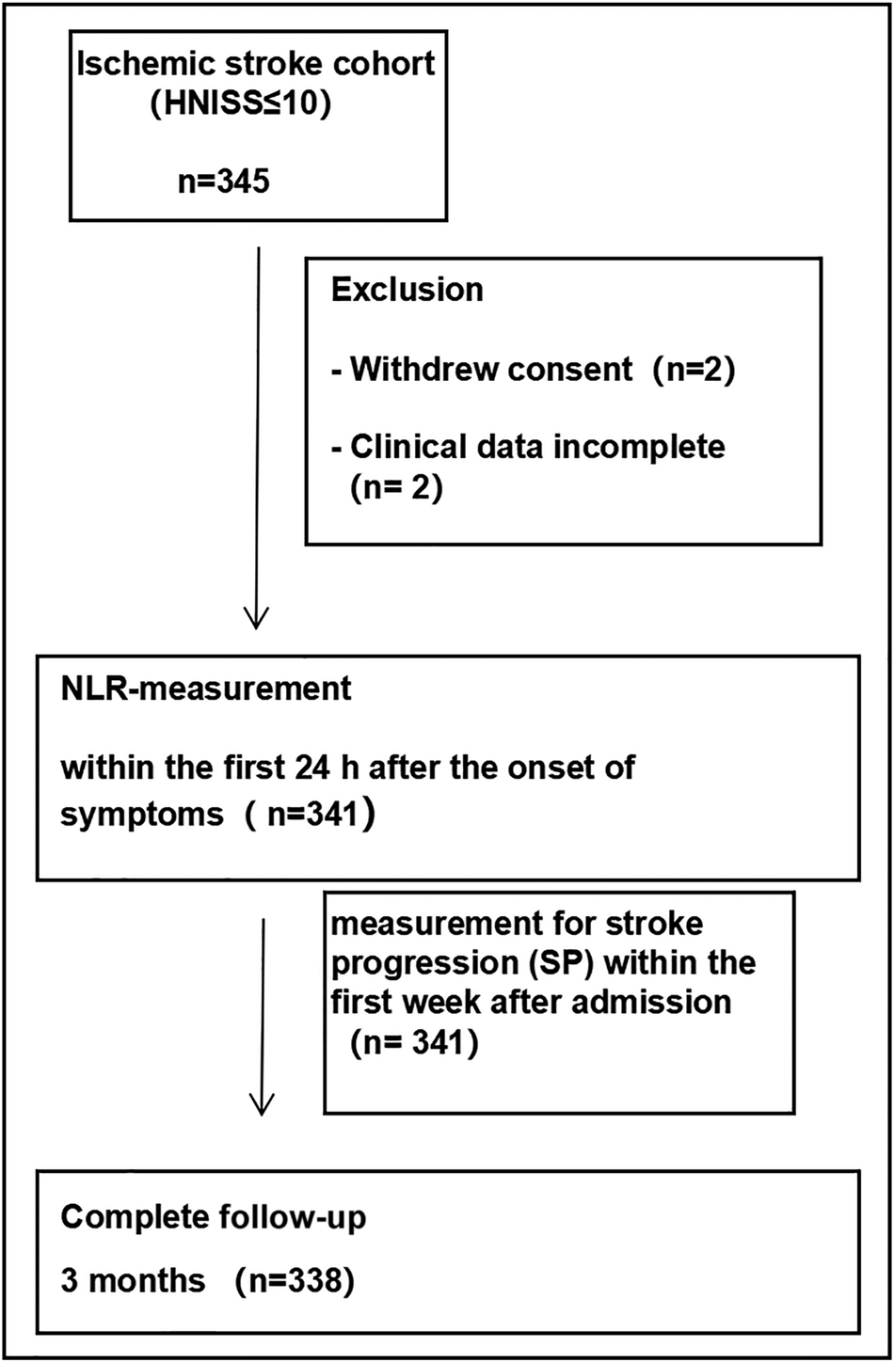
Flowchart illustrating patient enrollment/follow‐up and availability.

Exclusion criteria were as follows: (1) cases with MRI scanning contraindications; (2) hemorrhage transformation of infarct; (3) stent implantation and mechanical thrombectomy; (4) with pre‐stroke modified Rankin scale (mRS) score ≥2; (5) transient ischemic attack; (6) had significant liver or kidney dysfunction or cardiac impairment, the coexistence of other severe systemic diseases; (7) concurrent diagnosis of malignant neoplasms, active antitumor therapy; (8) other neurodegenerative disorders, or autoimmune conditions affecting the nervous system; and (9) unwillingness to participate in this study. The measurement of stroke severity was conducted by a certified member of the stroke team through the assessment of the on‐admission NIHSS score. The evaluation was subsequently performed daily during the patient's hospitalization. The examination of stroke etiology categorization was conducted using innovative criteria for subtype classification (Han et al., 2007). A CONSORT (Consolidated Standards of Reporting Trials) diagram of patients is shown in Figure 1.

The ethical board of the participating hospitals authorized this investigation. Before obtaining their agreement, all participants were first informed about the study. This research was registered in the Chinese Clinical Trial Registry with registration number ChiCTR2000029902.

### 2.2 Clinical parameters

Demographics (age and sex) and relevant vascular‐based risk parameter data were collected. We performed laboratory tests in a fasted state on the day of hospital arrival. Laboratory test indicators include total white blood cells, total white blood cell count, neutrophil count, lymphocyte count, monocyte (mmol/L), platelet count, HbA1c, fibrinogen (g/L), d‐dimer (mg/L), homocysteine (μmol/L), global cholesterol (mmol/L), triglycerides (mmol/L), low‐density lipoprotein (LDL)‐cholesterol (mmol/L), high‐density lipoprotein (HDL)‐cholesterol (mmol/L), together with NLR, LMR, monocyte‐to‐high‐density lipoprotein ratio (MHR), and PLR.

### 2.3 Outcome variables

The primary outcome was SP within the first week after admission. SP was defined as an incremental increase in the NIHSS score by ≥2 points in the total score within the first week after admission, excluding HT, edema, stroke recurrence, and a new infarct in another vascular territory. The NIHSS scale was evaluated daily, starting from the day of admission. The assessments were conducted within the same time frame each day, specifically in the morning, to minimize potential variations in patient conditions throughout the day. This method facilitated consistent and reliable monitoring of neurological status throughout the experiment.

Following their discharge, all patients were subjected to a post‐discharge follow‐up period of no less than 3 months, during which they were monitored by either a research nurse or a physician. All cases received conventional care following established protocols for stroke prevention (Kernan et al., 2014). The secondary outcome was the functional outcome 3 months after IS. Functional outcome was evaluated using the mRS. Functional outcome was determined through mRS scores, dichotomized for “favorable” (scoring 0–2) or better outcome and “unfavorable” or poor outcomes (scoring 3–6) (Banks & Marotta, 2007; Hacke et al., 1998).

### 2.4 Statistical analyses

SPSS 23.0 and GraphPad Prism 9.0 software were utilized, with continuous variables expressed as the mean ± standard deviation. The Kolmogorov‒Smirnov test was employed to ascertain the normality quality of the numerical variables. A single‐pair two‐tailed independent cohort *t‐*test was employed to analyze normally distributed data comparatively. Nonnormally distributed variables were assessed using the Mann‒Whitney test. Categorical variables were expressed as percentage rates and comparatively analyzed through the chi‐square test or (whenever predicted variable frequencies were minor) Fisher's exact tests. Univariate analysis was employed to evaluate clinical records and baseline profiles of cases, both with and without SP. The study utilized a multivariate logistic stepwise regression analysis to investigate the risk factors associated with SP. A poor outcome at 3 months of medical observation was the secondary outcome. The study performed a multivariate logistic stepwise regression analysis to investigate the associations between admission NLR levels and poor outcomes. The receiver operating characteristic (ROC) curve was analyzed to evaluate the NLR's ability to predict SP and a poor 3‐month outcome. A *p*‐value of <.05 was adopted to calculate the statistical significance of the datasets.

## 3 RESULTS

Out of the total sample size of 341 cases of acute IS, 56 patients (16.4%) were found to have SP, whereas the remaining 285 cases (83.6%) did not exhibit any SP. The incidence of diabetes mellitus was shown to be higher among individuals with IS who experienced SP within 7 days of hospitalization. In addition, total plasma cholesterol, initial NIHSS score, LDL cholesterol, and NLR were also significantly linked to SP by univariate analysis (Table 1). However, the two groups had no significant differences in the LMR, MHR, and PLR levels (*p* > .05, Table 1).

**TABLE 1:** Patient demographics and medical data.

Variable	SP group (*n* = 56)	No SP group (*n* = 285)	*p* Value
Age, years (means ± SD)	68.81 ± 13.0006	67.73 ± 11.818	.549
Male, *n* (%)	29 (51.7)	150 (53.2)	.908
Smoking (%)	20 (35.7)	110 (38.5)	.685
Hypertension (%)	37 (66.0)	183 (64.2)	.790
Diabetes mellitus (%)	20 (35.7)	56 (19.6)	.008^*^
Stroke history (%)	9 (0.16)	58 (20.35)	.461
WBC (10^9^/mL), median (IQR)	7.04 (2.48)	6.66 (2.84)	.216
Neutrophil (10^9^/mL), median (IQR)	4.915 (2.438)	4.490 (2.775)	.113
Initial NIHSS score, median (IQR)	4 (3)	3 (3)	.029^*^
Lymphocyte (10^9^/mL), median (IQR)	1.335 (1.08)	1.360 (0.845)	.579
Total plasma cholesterol (mmol/L) median (IQR)	4.215 (1.52)	4.550 (1.485)	.037^*^
Triglyceride (mmol/L), median (IQR)	1.240 (0.25)	1.420 (1.09)	.442
HDL‐cholesterol (mmol/L), median (IQR)	1.160 (0.26)	1.230 (0.42)	.443
LDL‐cholesterol (mmol/L), median (IQR)	2.270 (1.24)	2.57 (1.075)	.038^*^
Homocysteine (μmol/L), median (IQR)	11.30 (7)	11.95 (5.375)	.844
Monocyte (mmol/L), median (IQR)	0.37 (0.19)	0.41 (0.225)	.378
Fibrinogen (g/L), median (IQR)	2.535 (1.1275)	2.470 (0.865)	.117
HbA1c (means ± SD)	7.002 ± 1.7658	6.676 ± 1.7238	.181
NLR, median (IQR)	3.334 (4.864)	3.2137 (2.938)	.021^*^
LMR, median (IQR)	3.8775 (2.356)	3.194 (2.274)	.713
MHR, median (IQR)	0.306 (0.216)	3.287 (0.241)	.396
PLR, median (IQR)	113.73 (99.16)	118.66 (79.61)	.487
Treatment:			
Thrombolytic therapy, *n* (%)	13 (23.21)	48 (16.84)	.255
Single antiplatelet therapy, *n* (%)	25 (44.64)	151 (52.98)	.254
Dual antiplatelet therapy, *n* (%)	36 (64.29)	148 (51.93)	.090

Abbreviations: HDL‐cholesterol, high‐density lipoprotein‐cholesterol; LDL‐cholesterol, low‐density lipoprotein‐cholesterol; LMR, lymphocyte‐to‐monocyte ratio; MHR, monocyte to high‐density lipoprotein ratio; NIHSS, National Institute of Health Stroke Scale; NLR, neutrophil‐to‐lymphocyte ratio; PLR, platelet‐to‐lymphocyte ratio; SP, stroke progression.

^*^ *p* < .05 was considered statistically significant.

**FIGURE 2:**
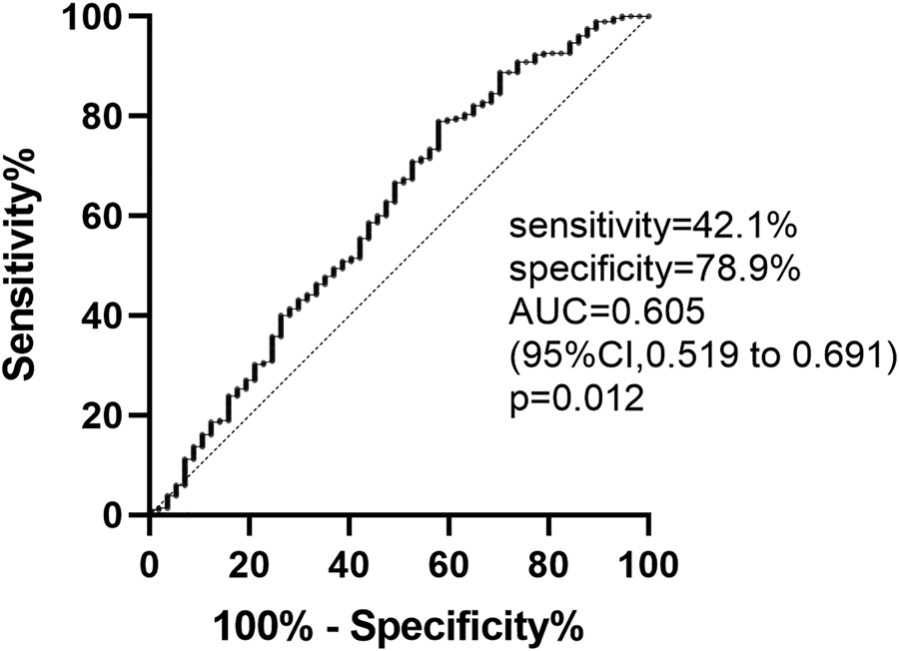
Receiver operating characteristics (ROCs) for neutrophil‐to‐lymphocyte ratio (NLR) in predicting stroke progression within ischemic stroke. AUC, the area under the curve; CI, confidence interval.

The mean NLR level of the total cases was 5.5534 ± 10.5238, with quartile levels as follows: 0.0526–2.1690 (first quartile), 2.1690–3.2528 (second quartile), 3.2528–5.3363 (third quartile), and 5.3363–179.714 (fourth quartile). Multivariate logistic stepwise regression analyses revealed that diabetes mellitus (adjusted OR .018, 95% confidence interval [CI], 1.145–4.185, *p* = .018) and NLR (adjusted OR 1.096, 95% CI, 1.037–1.157, *p* = .001) were independently linked with SP after adjusting for initial NIHSS score (Table 2). ROC was used to predict SP development probabilities inside IS situations using the NLR. The best threshold point was 5.421, with 42.1% sensitivity and 78.9% specificity. The area under the curve (AUC) was .605 (95% CI: .519–.691, *p* = .012) (Figure 2).

**TABLE 2:** Binary logistic regression for stroke progression analysis (adjusted by initial National Institute of Health Stroke Scale [NIHSS] score).

	*p* Value	OR	95% CI
diabetes mellitus	.003^*^	2.704	1.409–5.189
Total plasma cholesterol	.546	.798	.384–1.658
LDL‐cholesterol	.730	.842	.317–2.235
NLR	.001^*^	1.096	1.037–1.157

Abbreviations: 95% CI, 95% confidence interval; LDL‐cholesterol, low density lipoprotein‐cholesterol; NLR, neutrophil‐to‐lymphocyte ratio; OR, odds ratio.

^*^ *p* < .05 was considered statistically significant.

Among 341 cases, 338 completed 3 months of follow‐up. According to NLR quartiles, there were 83 cases within the first quartile cohort, 84 patients within the second quartile cohort, 86 patients within the third quartile cohort, and 85 subjects in the fourth quartile.

**FIGURE 3:**
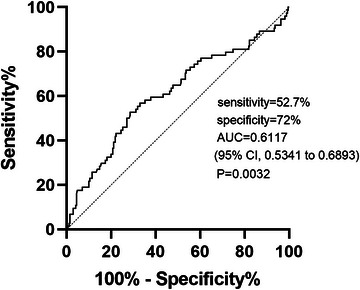
Receiver operating characteristics (ROCs) for neutrophil‐to‐lymphocyte ratio (NLR) for predicting 3‐month poor outcome within ischemic stroke. AUC, the area under the curve; CI, confidence interval.

Spearman rank‐order correlation showed that there was a significant association among NLR level, initial NIHSS, and mRS scores at 3 months (*p* < .001). Based on the results of a 3‐month follow‐up and dichotomies of mRS scores as mentioned, there were 13 (15.7%) cases in the first quartile cohort of NLR level with poor outcome, 11 (13.1%) cases in the second quartile, 17 (19.8%) cases within the third quartile cohort, and 26 (30.6%) in the fourth quartile. Binary logistic regression revealed that NLR (adjusted OR 1.071, 95% CI, 1.016–1.128, *p* = .010) was associated with poor functional outcomes after adjusting for the initial NIHSS score (Table 3). The ROC curve revealed that the best cut‐off for the NLR in predicting a poor 3‐month functional outcome was 4.2139, with a sensitivity of 52.7% and a specificity of 72%. The AUC was .6117 (95% CI, .5341–.6893, *p* = .0032) (Figure 3).

**TABLE 3:** Binary logistic regression for 3‐month poor outcome analysis (adjusted by initial National Institute of Health Stroke Scale [NIHSS] score).

	*p* Value	OR	95% CI
Diabetes mellitus	.755	1.116	.561–2.219
Total plasma cholesterol	.442	.758	.374–1.537
LDL‐cholesterol	.419	.677	.263–1.744
NLR	.010^*^	1.071	1.016–1.128

Abbreviations: 95% CI, 95% confidence interval; LDL‐cholesterol, low density lipoprotein‐cholesterol; NLR, neutrophil‐to‐lymphocyte ratio; OR, odds ratio.

^*^ *p* < .05 was considered statistically significant.

According to the new subtype classification (Han et al., 2007), the location and white matter classification (Fazekas scales) of IS were analyzed according to cases’ imaging data (Table 4). Among 167 patients of atherothrombosis with significant stenosis of a large artery, the mean HLR level was 4.3414 ± 3.976 within the SP cohort and 4.226 ± 3.102 within the No SP cohort. In 97 small artery disease cases, the mean HLR level was 5.5004 ± 5.499 within the SP cohort and 4.913 ± 4.370 within the No SP cohort. Furthermore, the mean HLR level was 5.645 ± 3.834 within the SP cohort and 3.845 ± 2.712 in the non‐SP cohort in 30 cardioembolism cases. No significant difference was found in NLR levels between both cohorts within the subtype classification (*p* > .05).

**TABLE 4:** Imaging classification of ischemic stroke.

	SP group (*n* = 56)	No SP group (*n* = 285)	*p* Value
New subtype classification			
ASLA (%)	29 (51.8)	138 (48.4)	.645
SAD (%)	16 (28.6)	81 (28.4)	.357
CE (%)	5 (8.9)	25 (8.8)	.970
SUD (%)	6 (10.7)	41 (14.4)	.466
Fazekas scales			
0 (%)	9 (16.1)	45 (15.8)	.958
1 (%)	25 (44.6)	131 (46.0)	.856
2 (%)	14 (25)	57 (20)	.400
3 (%)	8 (14.3)	52 (18.2)	.477
Stroke territory			
Anterior cerebral circulation (%)	39 (69.6)	175 (61.4)	.244
Posterior cerebral circulation (%)	17 (30.4)	110 (38.6)	.244

Abbreviations: ASLA, atherothrombosis with significant stenosis of a large artery; CE, cardioembolism; SAD, small artery disease; SP, stroke progression; SUD, stroke of undetermined etiology.

## 4 DISCUSSION

SP is a common event in short‐term IS cases (Weimar et al., 2005). In this study, approximately 16.4% of IS cases had SP within the first‐week postadmission. Compared with ranges from 12% to 42% within previous reports (Tei et al., 2000) and through Jørgensen et al.’s (1994) definition of SP, this definition of SP—based on an incremental increase in the NIHSS score—can accurately describe increases in severity or distribution.

Through multivariate logistic regression analyses, this study found a relationship between SP and diabetes mellitus. Although END and SP are defined differently, IS subjects with diabetes mellitus appear more likely to experience neurologic deterioration (Weimar et al., 2005). Based on current understanding, it is widely recognized that elevated levels of LDL cholesterol contribute to the progression of atherosclerosis. Prior research has indicated that cerebral atherosclerosis is IS's underlying etiological factor or precipitating circumstance (Banerjee & Chimowitz, 2017; Qureshi & Caplan, 2014). In addition, intracranial atherosclerosis and IS have high incidence and mortality rates in Asian populations (Johnston et al., 2009). Macrophages may interact with LDL cholesterol to expedite atherosclerotic plaque progression (Hamilton et al., 1999; Martens et al., 1998). This investigation found that in cases with IS, LDL‐cholesterol was unrelated to SP or with 3‐month poor outcomes. Although some scholars argue that upregulated LDL cholesterol is linked to a reduced risk of hemorrhagic stroke (Wang et al., 2013), a study by Lee et al. (2022) revealed that intensely lowering LDL‐C treatments were necessary to decrease the risk of stroke recurrence. Although elevated LDL has been associated with the onset of vascular disorders, it is unknown if it influences or worsens the prognosis of IS patients. In addition, our research revealed that SP and NLR levels significantly correlated. Although the ROC curve showed that the sensitivity of the NLR to diagnose SP was as low as 42.1%, the specificity was as high as 78.9%. Therefore, we believe that the predictive value of the NLR for SP cannot be ignored.

DeGraba et al. (1999) firmly hypothesized that the severity of the initial stroke would determine the progression of neurologic damage following acute IS. Diverging from their dichotomy of initial NIHSS score (≤7 and >7), we chose an incremental increase in NIHSS score ≥2 as the criteria for evaluating SP. This study also found that the initial NIHSS score correlates to early stroke development. In contrast to prior reports, the findings of this investigation demonstrated that homocysteine levels were not substantially associated with early stroke development (Kwon et al., 2014). One possible explanation is that their end‐point event is END, which differs from our definition of SP. Homocysteine levels did not predict IS functional impairment (Song et al., 2009). Based on these findings, more research is needed to confirm its association with the prognosis of IS.

Inflammatory reactions occurring at the blood–endothelium interface may be essential factors of ischemic injury in stroke (Huang et al., 2006). Oxygen radicals and proinflammatory mediators can enter the systemic circulation by destroying the brain–blood barrier or neurovascular units (Song et al., 2019). Neutrophils first infiltrate lesions and reach peak values within 24–72 h (Jin et al., 2013). Brain injury is aggravated by exacerbating oxidative stress (Chamorro et al., 2016). According to multiple investigations, there is a strong correlation between neutrophil levels and the severity and prognosis of cerebral infarction, mainly when the population of neutrophils is close to 5.8 > 103/μL, and the risk of cardiovascular events substantially increases (Avanzas et al., 2004). Although T cells are thought to be harmful during the progression of poststroke cerebral injury (Hurn et al., 2007), several T‐lymphocyte subtypes may have been considered to have protective effects, such as Th17 cells (Gillum et al., 2005; Korn et al., 2009). They appear to take on opposing roles. The clinical usefulness is also diminished if examined independently because the correlation between these subtypes may be disregarded.

The NLR, a noncomplex clinical biomarker easily obtained, can reliably predict the 3‐month functional results of patients with IS (Lux et al., 2020). Within this study, an NLR level of >4.2139 was determined to be a cut‐off point for poor outcomes within the IS, which was close to the results of Tokgoz et al. (2013) (NLR ≥ 5.0) and Brooks et al. (2014) (NLR ≥ 5.9). In addition, our study was identical to the results of Song et al. (2019) (NLR > 4). Hence, it may be concluded that an increased NLR appears to be a dependable indicator of unfavorable outcomes among individuals with IS during the intermediate‐term prognosis. Consequently, healthcare professionals must pay more attention to this factor in clinical settings.

We analyzed SP cohorts and No SP cohorts through imaging datasets of all included IS cases through stroke territory, stroke novel subtype classification, and Fazekas scales. The results showed that SP occurrence is unrelated to the novel stroke subtype classification. Several reports have suggested that the SP rate of cases in the posterior circulating stroke cohort is higher than that of patients in the anterior circulating stroke (ACS) cohort (Sumer et al., 2003). However, a few studies also report that SP is more common in ACS cohorts (Li et al., 2020). Therefore, it is controversial whether the location of IS continues to be a risk factor for SP. Additionally, based on the findings of this inquiry, it appears that neither the location nor the varieties of IS are associated with the occurrence of SP.

There are limitations to this investigation. The clinical records evaluated were received within 24 h after case admission, with such clinical data being variable during case admission. Therefore, it is still essential to consider whether the HLR level changes after admission for therapeutic reasons. In addition, this was a two‐center study, and a multicenter/multiethnic case analysis should be carried out. Third, such research participants included cases with thrombolytic therapy. Although there was no significant difference between the SP group and the no SP group in the primary treatment methods, there was still a risk of bias in results concerning shifts in the NIHSS score for thrombolytic therapy. Therefore, it is strongly recommended that future research endeavors investigate the comparative outcomes of thrombolytic treatment and medical therapy.

## 5 CONCLUSION

This investigation revealed that diabetes mellitus and the NLR were significantly associated with SP. High levels of NLR can be a significant predicting tool for 3‐month poor outcomes within IS cases.

## AUTHOR CONTRIBUTIONS

Chongxi Xu and Linrui Cai conceived the study and designed the protocol. Xingyang Yi and Tong Yi collected all patient data. Yu Hu analyzed the data. Chongxi Xu wrote the first draft of the paper. Linrui Cai and Yu Hu reviewed and revised the manuscript. All authors critically revised successive drafts of the paper and approved the final version. Yu Hu and Chongxi Xu are the study guarantors. All authors read and approved the final manuscript.

## CONFLICT OF INTEREST STATEMENT

The authors declare that they have no conflicts of interest.

### CONSENT TO PARTICIPATE

Written informed consent was obtained from all subjects before the study.

### CLINICAL TRIAL REGISTRATION

This study was registered in the Chinese Clinical Trial Registry (Registration number: ChiCTR2000029902). Date 2020/02/16. http://www.chictr.org.cn/index.aspx.

### PEER REVIEW

The peer review history for this article is available at https://publons.com/publon/10.1002/brb3.3261.

## Data Availability

All data generated or analyzed during this study are included in this published article. Further inquiries can be directed to the corresponding author.
